# Valorization of Yarrow (*Achillea millefolium* L.) By-Product through Application of Subcritical Water Extraction

**DOI:** 10.3390/molecules25081878

**Published:** 2020-04-18

**Authors:** Jelena Vladić, Martina Jakovljević, Maja Molnar, Senka Vidović, Milan Tomić, Zorica Drinić, Stela Jokić

**Affiliations:** 1 Faculty of Technology, University of Novi Sad, Bulevar cara Lazara 1, 21000 Novi Sad, Serbia or vladicj@uns.ac.rs (J.V.); senka.vidovic@uns.ac.rs (S.V.); 2 Faculty of Food Technology, J. J. Strossmayer University of Osijek, Cara Hadrijana 10E, 31000 Osijek, Croatia; martina.jakovljevic@ptfos.hr (M.J.); drinic_zorica@yahoo.com (Z.D.); stela.jokic@ptfos.hr (S.J.); 3 Faculty of Agriculture, University of Novi Sad, Trg Dositeja Obradovica 8, 21000 Novi Sad, Serbia; milanto@polj.uns.ac.rs

**Keywords:** *Achillea millefolium*, yarrow, subcritical water, polyphenols, antioxidant activity, optimization

## Abstract

In the present study, valorization of yarrow (*Achillea millefolium*) by-product from the filter tea industry was investigated through the application of subcritical water for the extraction of bioactive compounds. The influence of different process parameters (temperature 120–200 °C, extraction time 10–30 min, and HCl concentration in extraction solvent 0–1.5%) on extract quality in terms of content of bioactive compounds and antioxidant activity was investigated. Optimal conditions of the extraction process (temperature 198 °C, extraction time 16.5 min, and without acidifer) were determined and, when applied, the most efficient exploitation of by-products is achieved, that is, attainment of extracts rich in total phenols and flavonoids and high antioxidant activity. In addition, by applying the high performance liquid chromatographic analysis, the content of chlorogenic acid was determined as well as the hydroxymethylfurfural content in obtained extracts. The results demonstrated that subcritical water can be successfully used for utilization of yarrow by-products for obtaining extracts rich in antioxidants.

## 1. Introduction

Yarrow (*Achillea millefolium* L.) as a widespread plant coming from the Asteraceae family has a long history of use, beginning in ancient times when it was used as a wound-healing remedy. Apart from that, the traditional use of yarrow includes treatment of inflammatory and respiratory infections, as well as spasmodic gastro-intestinal and hepato-biliary disorders [1,2,3]. Owing to its long tradition of use, *A. millefolium* is often used in various industrial tea mixtures, as well as in phytopharmaceuticals [2,4,5]. The reason for the frequent use of *A. millefolium* lies precisely in its composition, which includes phenolic compounds such as flavonoids and phenolic acids, which are well known for their numeorous pharmacological effects [6] including antitumor, antimicrobial, anti-inflammatory, and antioxidant properties [3,7,8].

Medicinal plants are most commonly used in the form of herbal tea [9], individually or in a tea blend. In the process of the production of filter tea, very small particles, known as herbal dust, are formed owing to grinding of plant material [10]. As particles of herbal dust are smaller (<0.315 mm) in regards to particles of filter paper, this fraction cannot be used in the form of filter tea and is considered as a by-product. Although a certain loss of bioactive components is noticed during the production of herbal tea, herbal dust still represents a good source of bioactive components [9]. This has already been confirmed in several studies in which, with various extraction techniques, extracts with significant amounts of bioactive components from herbal dust were obtained [10,11,12]. Additionally, it was confirmed that yarrow by-product represents a very good source of bioactive compounds. In a previously published study, the conventional maceration process with mixture ethanol/water was applied for obtaining a liquid extract of yarrow by-products, which is further transformed in dry form via spray drying [13].

Conventional techniques of extraction are being replaced with green extraction technologies, which provide a range of advantages compared with conventional ones, such as ecological benefits owing to the absence of toxic solvents, economic benefits in terms of achieving higher process efficacy and product quality in a short time period, and absence of the product purification step. Subcritical water extraction (SWE) is one of the green technologies thatuses water in subcritical state, which is achieved at temperatures between 100 and 374.15 °C, and at the pressure high enough to keep the water in the liquid state. At ambient temperature and atmospheric pressure, water has one of the highest dielectric constants (i.e., ε = 80 at 25 °C), and in that state, it is suitable for the extraction of highly polar compounds [14], which also makes it inadequate for the extraction of low-polarity compounds [15]. However, in subcritical state, water polarity decreases with a dramatic drop in dielectric constant caused by the increase in temperature. For example, when the temperature and pressure increase to 250 °C and 50 bar, respectively, the dielectric constant drops to ε = 27, making it similar to organic solvents such as methanol (ε = 33), ethanol (ε = 24), acetone (ε = 20.7), and acetonitrile (ε = 37). Under such conditions, the water will behave similarly to organic solvents, dissolving a wide spectrum of components with low and medium polarity [16]. Also, owing to the lower viscosity and higher diffusivity of subcritical water, diffusion into the plant matrix and the release of compounds from the solid to liquid phase is much more efficient [17].

In SWE, temperature represents the most significant influencing factor because an increase in temperature leads to better mass transfer between the matrix and media, solubility of solutes, and diffusion rate at a lower solvent viscosity and surface tension [18,19,20,21]. Extraction time is also an important factor that can significantly impact the quality of extracts, because long-term exposure to high temperature can cause degradation of bioactive components or formation of new antioxidants [20,22,23]. Furthermore, to secure as feasible a process as possible, it is necessary to optimize the process conditions in terms of minimal energy consumption and operational costs. Moreover, the availability of polyphenols in solvents is different depending on the characteristics such as type of plant material and fragmentation, because they can be found to be bound via ester linkages with polysaccharides in the cell wall [24]. Therefore, by adding an acidifier and using hydrolysis, the extraction of bioactives can potentially be aided.

To determine the most efficient and feasible process of extraction, which entail a high content of bioactive components and biological potency of extracts with minimal production costs as the end results, it is necessary to conduct the process of optimization of parameters. The optimization can be achieved by applying the traditional one-variable-at-a-time technique, which includes monitoring the influence of one factor on desired responses while other factors remain constant. This approach includes a high number of experiments, which is time-consuming and alsoresults in increased costs. Additionally, as the interactive effects between the investigated factors are not monitored, it does not provide a complete insight into the impact of the parameter on the responses. The set of mathematical and statistical tools thatenable the determination of impact of several factors on a set of responses and simultaneously provide the optimization of investigated variables to achieve the best system performance is the response surface methodology (RSM) [25].

Each material possesses unique characteristics, which is the reason it is not possible to define a single extraction strategy that could be applied to all cases. Therefore, to achieve the maximum benefits regarding product quality, waste management, production process management, and exploitation of natural resources, it is necessary to conduct an optimization study for each individual material.

Considering the aforementioned, the main objective of this research was to apply SWE in order to obtain extracts of *A. millefolium* by-product with a high yield of polyphenols and high antioxidant activity. Furthermore, the quality of extracts was also monitored in terms of the content of chlorogenic acid and content of hydroxymethylfurfural (HMF) as an indicator of hydrolyse. To the best of our knowledge, this is the first study that investigates a green optimization process of yarrow by-product sourced from the industry and represents a contribution to green growth and the circular economy.

## 2. Results and Discussion

In order to estimate the applicability of SWE for extracting phenolic compounds from the herbal dust of *A. millefolium*, the SWE extraction was performed at different process parameters defined by Box–Behnken experimental design (BBD) (Table 1).

The experimental design was set based on previously published studies. According to Plaza and Turner [26], the application of higher temperatures causes the extraction of more contaminants, which further leads to lower selectivity and a potential increase in costs because of the additional purification step. It was observed that a temperature increase from 120 to 170 °C provides a higher yield of phenols extracted from *A. uva-ursi* herbal dust. Furthermore, higher temperatures led to a drop in the content of phenols [27]. The same findings were recorded by Singh and Saldana [28], who discovered that a higher yield of phenolics was extracted from potato peel at temperatures ranging from 140 to 180 °C. A further rise in temperature up to 240 °C affected the efficiency of the extraction and produced a lower yield of phenols [28]. In addition, in a previous study in which winter savory, purple coneflower, and comfrey root were used as a sources of polyphenols, the optimal SWE process parameters were as follows: 200 °C and 20.8 min [20]; 148 °C and 17.8 min [29]; 200 °C, 25.6 min, and 0.0075% HCl [21], respectively.

It was demonstrated that extended exposure of material to higher temperatures can lead to the development of brown coloring and degradation owing to the formation of products of caramelization and Mailard reactions [17,26]. On the other hand, the results of other studies suggest that by providing acidic conditions, these reactions can be decreased. According to Euterpio et al. [30], a significant improvement in the extraction yield was noted when the pH was set to a very low value by adding modifier. The solubility of the compounds of interest can be improved by adding certain modifiers and can also impact the material’s physical characteristics and the desorption of the compounds from the material [26]. The extraction of anthocyanins from red cabbage was promoted by adding 1% formic acid in water [31]. Additionally, the favourable impact of acidic conditions of SWE on phenols can be explained by the retained stability of isolated phenols and prevented enzymatic oxidation of phenols. Moreover, polyphenols can frequently be found in the form of glycosides and, to obtain free aglycones, these components have to be hydrolyzed, which can be achieved with acid-catalyzed or enzyme-catalyzed hydrolysis. With the application of SWE, this process can potentially be provided without the use of a catalyst [22,32]. Nevertheless, severe conditions such as extended exposures to high temperatures can represent the cause of a drop in extraction efficiency. Therefore, optimization of the process of extraction of target compounds is necessary. In order to potentially improve the efficiency of extraction of bioactives and potential hydrolysis and the release of polyphenolic compounds from glycosides with minimal formation of undesired components and attainment of extracts rich in polyphenols, this study investigated the parameters in the following ranges: temperature 100−200 °C, extraction time 10−30 min, and HCl concentration 0–1.5%.

In obtained extracts, the contents of total phenols (TPH) and total flavonoids (TFL) were determined, as well as antioxidant activity (Table 2). In addition, extracts were analyzed by high performance liquid chromatographic method (HPLC) to determine individual components present in extracts.

### 2.1. Determination of Total Phenols Content

Experimentally obtained values of TPH in SWE extracts of *A. millefolium* by-product varied from 54.54 to 128.20 mg GAE/g (Table 2), depending on the applied extraction parameters. The lowest TPH content was obtained at a temperature of 120 °C, time of 30 min, and HCl concentration of 0.75%, while the highest TPH content was obtained at a temperature of 200 °C, time of 20 min, and without HCl. Vitalini et al. [33] reported a significantly higher TPH content of 281.7 mg/g in methanolic *A. millefolium* extract. However, in the same study by Vitalini et al. [33], yarrow aerial parts were used as plant material and not industrial by-products. Additionally, yarrow extracts were obtained by extraction with *n*-hexane and methanol in a Soxhlet apparatus, which has numerous ecological and economical disadvantages.

In another study [34], a lower TPH content was observed in a methanolic extract (123.9 ±2.6 mg GAL/g), which was very similar to the one achieved by SWE at higher temperatures in this study. On the other hand, the TPH content was even lower in the aqueous extract (48.4 ± 2.7 mg GAE/g), similar to the content obtained by SWE at lower temperatures, which best illustrates how properties of water as a solvent change by increasing temperature, that is, maintaining subcritical state. Moreover, similar results of TP content obtained at a higher temperature were determined in methanolic extracts (128.36 mg/g), while in infusion and decoction, the values were lower (112.28 ± 0.22 and 43.66 ± 0.57 mg/g) [3].

In a study by Vladic et al. [13], where the same plant material (herbal dust) was used for investigation, the content of TPH in powder obtained using spray drying was notably higher than in extracts obtained with subcritical water (169.41 mg GAE/g SE). Initially, liquid extract of yarrow was obtained by the maceration procedure under the following extraction conditions: room temperature, solvent 50% mixture of ethanol/water, and extraction time of five days. For determination of the content of TPH and TFL, the obtained powder was dissolved in methanol (extraction time 24 h) and the obtained extracts were used for determination of polyphenols content. Therefore, the conventional extraction method is more suitable for valorization of yarrow by-product. However, having in mind the requirements of the industry in terms of a fast green production process, a long extraction time (five days) is not feasible.

**Figure 1:**
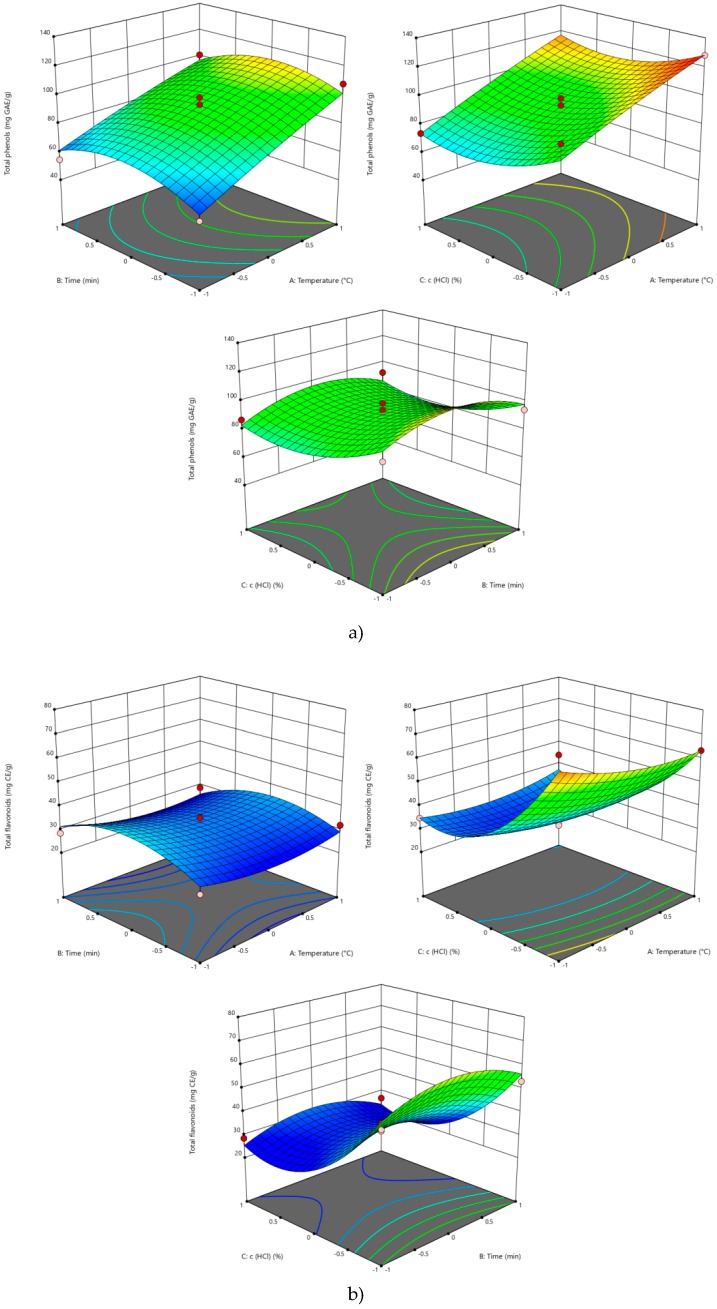

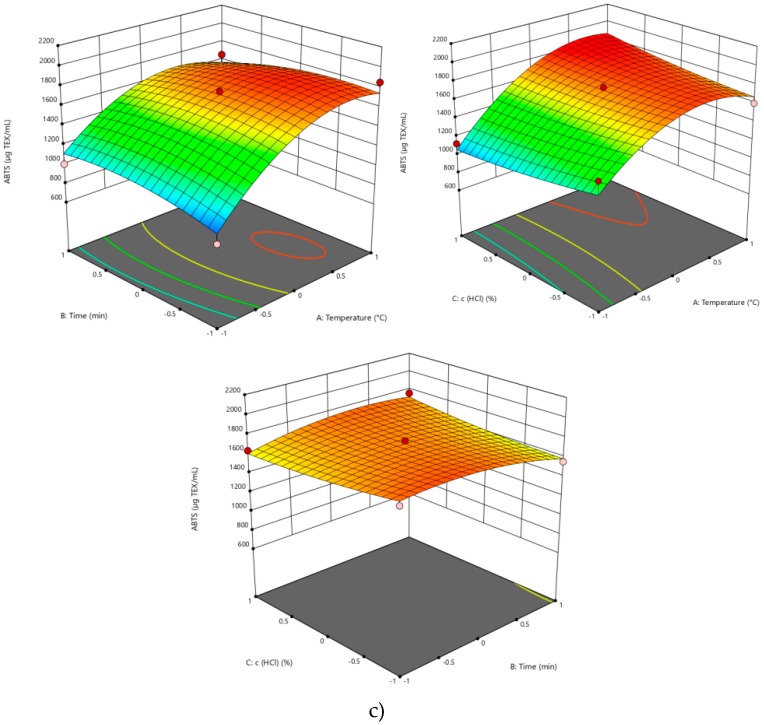
Three-dimensional plot for the obtained responses of (**a**) total phenols (TPH), (**b**) total flavonoids (TFL), and (**c**) antioxidant activity as a function of extraction temperature, time, and HCl concentration.

According to the obtained results (Table 3 and Figure 1), only the temperature of extraction had a statistically significant influence on the TPH content, while the extraction time and addition of HCl did not influence this investigated response. TPH content was increased with the increase of temperature owing to the increased polyphenol solubility and mass transfer coefficient between the matrix and media [18,19]. Higher temperatures consequentially led to lowering surface tension, which further allows better pore filling and solubilization of phenols [21,35]. Similar findings, which state that only temperature exhibits an important influence, were determined in studies where SWE was used for the extraction of bioactive compounds from *Satureja montana, Symphytum officinale, Coriandrum sativum,* and *Allium ursinum* [20,21,36,37].

Previous studied report that HCl can have a highly important positive or negative impact on the extraction of TPH [27,38,39]. In present study, pressure exerted an insignificant impact. Similar findings were determined in a study by Vladic et al. [21], where HCl addition did not demonstrate a significant effect on TPH yield, which was also the case in a study by Pavlic et al. [12], where that effect was only moderately significant. Finally, the efficiency of the recovery of phenolics depends on the nature of the material and their distribution in matrix, as well as stability. This points to the importance and necessity of the optimization of extraction of target compounds [33].

### 2.2. Determination of Total Flavonoids Content

TFL content in *A. millefolium* extracts ranged between 26.32 and 79.19 mg CE/g (Table 2), depending on the applied parameters. The lowest TFL content was obtained in the extract obtained at a temperature of 160 °C, time of 30 min, and HCl concentration of 1.5%, while the highest TPH content was achieved at a temperature of 120 °C, time of 20 min, and without HCl. These values are higher than those obtained in the study by Eghdami and Sadeghi [34], where TFL content in methanolic and aqueous extract was 41.2 ± 1.7 and 13.15± 1.8 QE/g, respectively. In a paper by Dias et al. [3], lower values of TFL were measured in methanolic extract, infusion, and decoct (24.56 ± 0.36, 22.96 ± 0.10, and 11.14 ± 0.05, respectively). However, like in the case of TPH, the content of TFL in dry extracts of yarrow by-products (obtained by maceration with ethanol/water followed by spray drying and methanol extraction) was significantly higher, at 113.08 mg CE/g SE [13].

As presented in Figure 1 and Table 3, the addition of acidifier significantly influenced the TFL content (*p*-value 0.004) in obtained extracts. The TFL content was increased with the decrease of HCl concentration, which is in accordance with the literature [40], as it is demonstrated that HCl may hydrolyse acylated flavonoids, which consequently leads to a lower concentration of flavonoids.

The extraction time and temperature of the process did not exert a significant effect on TFL separation from yarrow by-products, while the quadratic terms of extraction time and concentration of HCl exhibited a significant influence on TFL. As suggested by Singh and Saldana, the influence of SWE temperature differs depending on the material and the concentration of the bioactives compounds [28]. Munir et al. [22] reported a similar impact of temperature, which had no significant effect on TFL, while pH had a significant impact on TFL. Moreover, a negligible impact of extraction time had been noted in the extraction of *S. montana* [20]. Temperature causes the disruption of material, however, increasing the temperature above the optimal values could cause degradation of bioactive constituents [27].

### 2.3. Determination of Antioxidant Activity

The values obtained for antioxidant activity varied between 890.92 and 1853.57 µg TEX/mL (Table 2). The lowest antioxidant activity was obtained at a temperature of 120 °C, time of 10 min, and HCl concentration of 0.75%, while the highest antioxidant activity content was obtained at a temperature of 200 °C, time of 10 min, and HCl concentration of 0.75%. Antioxidant activity can be determined by different tests with different principles that do not give the same results, and for this reason, it is difficult to make a concrete comparison of the results. For instance, the antioxidant activity may be presented as percent inhibition of DPPH, with those for methanol and water extract being 75 and 50.8%, respectively [34]. For the DPPH assay, the results may also be presented as EC_50_, with those for methanolic extract, infusion, and decoct being 0.50, 0.40, and 0.25 mg/mL, respectively, in wild yarrow samples, while in extracts of commercial samples prepared in the same way, the results of EC_50_ were slightly lower (0.37, 0.22, and 0.20 mg/mL, respectively). In addition to DPPH assay, a few other assays for antioxidant activity were applied on the same extracts, confirming that the results depend on the assay applied and the way of expressing the results [3].

The results presented in Table 3 and Figure 1 demonstrate that antioxidant activity was significantly affected by the temperature of extraction, as well as the quadratic term of temperature. Using the Pearson′s correlation, a positive correlation was shown between the antioxidant activity and TPH (*p-*value 0.001), while the correlation with TFL was not noticed. Other parameters as well as interactions between them have no significant effect (*p*>0.05) on the antioxidant capacity of extracts.

Temperature had a similar impact on the antioxidant activity; therefore, the antioxidant activity was increased with the increase of temperature, which was reported in various studies [20,21,41]. The antioxidant activity of comfrey root extracts significantly increased with the increase in extraction temperatures from 120 to 200 °C, which correlated positively with the content of polyphenols [21]. Additionally, a similar impact of temperature was established in SWE of deodorized thyme [42] and pomegranate seed residue extracts [43], where the strongest and highest antioxidant activities were measured when the extraction was performed at a temperature between 180 and 240 °C. The high antioxidant activity of extracts acquired at such a relatively high temperature may have been related to the formation of new bioactive compounds during the extraction process via Maillard reactions [42].

### 2.4. Analysis of Variance (ANOVA)

The estimated coefficients of second-order response models for the TPH and TFL content and the antioxidant activity based on the BBD are given in Table 3. According to the ANOVA results presented in Table 4, the models for all investigated responses were statistically significant (*p* ≤ 0.05). In addition, error analysis showed a non-significant lack of fit (*p* = 0.0753–0.2002), indicating that the second-order polynomial model could be used to effectively present the relationship between the parameters. Additionally, determination coefficients (R^2^) for the TPH content, TFL content, and antioxidant activity values (0.9044, 0.9562, and 0.9175, respectively) indicate that the applied model is well-fitted to the experimental results.

With the purpose of providing the optimal quality of extracts in terms of content of polyphenols and antioxidant capacity, the optimization of parameters was performed and the extraction conditions were determined (temperature 198 °C, extraction time 16.5 min, and without acidifer). When the established parameters are applied, they provide the optimal quality of extracts (TPH 117.966 mg GAE/g, TFL 62.8345 mg CE/g, and antioxidant activity 1730.84 µg TEX/mL), which was confirmed experimentally.

Furthermore, the optimization was performed in a way that the extraction parameters were minimized (lowest temperature, shortest time of extraction, and minimal addition of acidifier) with simultaneous attainment of the maximal/optimal quality of extracts. In this case, by using conditions of temperature of 151.2 °C during a period of 11.1 min without acidifier, extract with TPH of 94.079 mg GAE/g, TFL of 59.55 mg CE/g, and antioxidant activity of 1691.71 µg TEX/mL was obtained.

### 2.5. Chemical Composition of Extracts Determined by HPLC-UV

The composition of the extracts was analyzed by HPLC in order to attempt to elicit individual components present in larger quantities. As presented in Table 5, HMF was detected in almost all samples except in those obtained at higher temperatures, while chlorogenic acid was detected only in a few samples. HMF, as a compound formed through Maillard and/or caramelization reactions, was present in most processes of the thermal processing of food, especially in carbohydrate-rich foods [44,45]. As HMF is a component with known cytotoxic, mutagenic, carcinogenic, and genotoxic effects [44,46], its presence in finished products is not desirable.According to Herrero et al. [47], an increase in extraction temperature in the range from 125 to 200 °C leads to greater formation of HMF, especially at a temperature of 200 °C. However, in our study, at a temperature of 200 °C and in the presence of different concentrations of HCl (0.75% and 1.5%), HMF was not detected in the samples, which is a possible result of degradation, polymerization, and reaction with other compounds [48]. Only in the sample obtained at 200 °C and without acid, a certain amount of HMF was detected. This trend was also observed in a paper by Tomšik et al. [30]. The highest amount of HMF was achieved at a temperature of 160 °C, an extraction time of 20 min, and a HCl concentration of 0.75%. Moreover, the correlation between the HMF content and antioxidant activity in obtained extracts was not determined.

In addition, the chlorogenic acid as a dominant compound in the phytochemical profile of *A. millefolium* [3,33] was also detected in several samples. It was recorded that higher temperatures of extraction are not favourable for the chlorogenic acid, so in extracts obtained at the highest temperature of 200 °C, chlorogenic acid was not detected in the samples. At the middle temperature, one combination thatwas the most adequate for obtaining extracts with chlorogenic acid was 160 °C, the shortest time of exposure, and without acididifer. Meanwhile, the lowest temperature of 120 °C used was adequate for obtaining extracts with chlorogenic acid, except in the case when HCl was used in a concentration of 1.5%. Therefore, it can be concluded that chlorogenic acid in extract of yarrow can be extracted by applying lower temperatures and optimal conditions of 120 °C, 20 min, and without acidifier.

In samples obtained at a lower temperature (120 °C), without HCl, and with 0.75% HCl, 21.2−30.4 µg/mL of chlorogenic acid was detected, whereas in the sample with 1.5% HCl, chlorogenic acid was not detected. Moreover, in one sample obtained at 160 °C without HCl, chlorogenic acid was detected in a concentration of 16.8 µg/mL. These results are similar to those obtained with 40% methanol, where the content of chlorogenic acid was 0.34%−2.75% in dried plant material [6].

## 3. Materials and Methods

### 3.1. Chemicals and Reagents

Folin–Ciocalteu reagent, (±)-catechin, 2,2′-azino-bis(3-ethylbenzothiazoline-6-sulfonic acid) diammonium salt (ABTS), and Trolox (6-hydroxy-2,5,7,8-tetramethylchroman-2-carboxylic acid) were purchased from Sigma-Aldrich (Sternheim, Germany). Gallic acid was purchased from Merck (Darmstadt, Germany). Potassium persulfate (99% pure) was obtained from Acros Organics (Geel, Belgium). All other reagents used were of analytical grade.

### 3.2. Plant Sample

Plant material *A. millefolium* used in this research represents the by-product of a herbal filter tea factory. The investigated herbal dust is created during the process of cutting, grinding, and fractionating of raw material in the herbal filter tea factory. The material was collected in the local herbal tea factory Fructus, Backa Palanka, Serbia and stored in paper bags at room temperature.

### 3.3. Subcritical Water Extraction

SWE was carried out using a batch-type high-pressure extractor (Parr Instrument Company, Moline, IL, USA). The extraction procedure and apparatus were described previously [37]. The extractor was filled with 10 g of *A. millefolium* herbal material and 100 mL of double-distilled water. The independent variables for extraction were temperature (120−200°C), extraction time (10−30 min), and concentration of HCl (0%−1.5%), while pressure was held constant (30 bar). The obtained extracts were filtrated through filter paper under vacuum and stored at 4 °C in a dark place until analysis.

### 3.4. Experimental Design

Response surface methodology was employed to study the effect of independent variables temperature (X_1_, 120–200 °C), extraction time (X_2_, 10–30 min), and HCl concentration (X_3_, 0–1.5%) on the TPH content, TFL content, and antioxidant activity as responses. The Box–Behnken experimental design was selected to propose the model for the investigated responses. The experimental design consisted of fifteen trials in random order, including three replicates at the central point. Table 1 shows the three independent variables encoded to three levels (−1, 0, and 1).

A second-order polynomial model (Equation (1)) was fitted to results in order to correlate the relationship of each factor to the response [25]:

Y = β_0_ + ∑β_i_X_i_ + ∑b_ii_X_ii_^2^ + ∑b_ij_X_i_X_j_ (1)

where Y represents the response variable; X_i_, X_ii_, and X_i_X_j_ represent the linear, quadratic, and interactive terms of the coded independent variables, respectively; and β_0_, β_i_, β_ii_, and β_ij_ are the regression coefficients for intercept, linearity, quadratic, and interaction intercept terms, respectively.

Analysis of variance (ANOVA) was carried out to identify the adequacy of the developed model with the significance levels of 0.05 (significant) and 0.10 (moderately significant). The coefficient of multiple determination (R^2^), coefficient of variance (CV), *p*-values for the model, and lack of fit coefficient were obtained from analysis of variance. The statistical software Design-Expert v.7 Trial (Stat-Ease, Minneapolis, MN, USA) was employed for the all computation and graphics in this study.

### 3.5. Determination of Total Phenols and Total Flavonoids Content

Total phenols (TPH) content in obtained extracts was measured according to the Folin–Ciocalteu procedure described by Kähkönen et al. [49]. Absorbance was measured at 750 nm (6300 Spectrophotometer, Jenway, Staffordshire, UK). Calibration curve was defined using gallic acid as standard and the results were expressed as mg of gallic acid equivalents (GAE) per g of extract. To determine the TFL content, the aluminum chloride colorimetric assay was used [50]. Absorbance was measured at 510 nm. TFL content in plant extracts was expressed as mg of catechin equivalents (CE) per g of extract through the standard calibration curve defined with catechin.

### 3.6. Determination of Antioxidant Activity

The antioxidant activity of *A. millefolium* extracts was estimated using the Trolox equivalent antioxidant capacity (TEAC) assay previously described by Miller et al. [51] with some modifications. ABTS and potassium persulfate were dissolved in distilled water to a final concentration of 7 mM and 2.45 mM, respectively. These two solutions were mixed and the mixture was left in a dark space at room temperature for 12 h before further use in order to produce ABTS+. In this study, the ABTS+ solution was diluted with distilled water to an absorbance of 0.700 ± 0.02 at 732 nm. Samples or Trolox standards (final concentration 10–150 μM) were added to diluted ABTS+ solution and the absorbance was measured 3 min after mixing using a spectrophotometer (Agilent Cary 60 UV/vis, Aligent Technologies, Santa Clara, CA, USA). The results were expressed as mg Trolox equivalents per mL of extract (mg TEX/mL).

### 3.7. HPLC Analysis

Determination of HMF and chlorogenic acid was performed using RP-HPLC (reversed phase-high performance liquid chromatographic method) method with UV detection, on Cosmosil 5C18-MS-II column (Nacalai Tesque, Inc., Kyoto, Japan), 150 mm long with an internal diameter of 4.6 mm. The analysis was performed with gradient elution with acetonitrile as phase A and 1% aqueous formic acid as phase B, at room temperature, with a flow rate of 1.0 mL/min, injection volume of 20 µL, and UV detection wavelength of 280 nm. Gradient conditions were: 0−9 min 5%−20% of A, 9−10 min holding 20% of A, 10−15 min 20%−5% of A, with an equilibration time of 10 min. The stock solutions of HMF and chlorogenic acid standard were prepared in a solvent and calibration was obtained at eight concentrations (concentration range 10.0, 20.0, 30.0, 50.0, 75.0, 100.0, 150.0, and 200.0 mg/L). Linearity of the HMF calibration curve was confirmed by R^2^ = 0.9996 with limit of detection (LOD) of 1.79 mg/mL, limit of quantification (LOQ) of 5.9 mg/mL, and HMF retention time of 10.7 min. Linearity of the chlorogenic acid calibration curve was confirmed by R^2^ = 0.9998 with limit of detection (LOD) of 0.016 mg/L, limit of quantification (LOQ) of 0.054 mg/L, and retention time of 14.3 min.

## 4. Conclusions

In the present study, yarrow by-product/herbal dust was successfully used for the extraction of bioactive components by SWE. The impact of extraction parameters (temperature, extraction time, and added acidifier) on TPH and TFL content and antioxidant activity determined by TEAC assay was evaluated. According to the statistical analysis, only temperature of extraction had a statistically significant influence on the TPH content and antioxidant activity, while the TFL content was significantly influenced only by added acidifier. Considering that extraction time showed no significant influence on the responses, a shorter extraction time is desirable from the economic point of view. In the obtained extracts, HMF as Maillard product indicator and chlorogenic acid as the main phenolic compound were determined by HPLC. HMF was detected in almost all samples, except in those obtained at higher temperatures in which it ranged between 5.8 and 39.3 µg/mL of extract. Chlorogenic acid was detected only in samples obtained at lower temperatures. Having in mind that this study provides practical and applicable results and a green technological path of valorisation of food by-products/waste, this represent a potential basis for the circular economy as well as a significant contribution to the protection of the environment.

## Figures and Tables

**Table 1:** Coded and actual levels of independent variables for the designed experiment.

Independent Variable	Symbol	Level		
		Low (−1)	Middle (0)	High (+1)
Temperature (°C)	X_1_	120	160	200
Time (min)	X_2_	10	20	30
HCl concentration (%)	X_3_	0	0.75	1.5

**Table 2:** Content of total phenols (TPH), total flavonoids (TFL), and antioxidant activity (expressed as ABTS ^1^ activity).

Run	X_1_[T]	X_2_ [t]	X_3_ [c(HCl)]	TPH	TFL	ABTS
	[°C]	[min]	[%]	[mg GAE/g] ^2^	[mg CE/g] ^3^	[µg TEX/mL] ^4^
1	0	−1	1	86.977	28.48	1637.90
2	−1	0	−1	101.67	79.19	1410.60
3	−1	−1	0	52.42	27.29	890.92
4	0	0	0	98.64	35.17	1754.68
5	0	0	0	86.77	32.57	1665.47
6	1	−1	0	107.90	31.94	1853.57
7	1	1	0	101.08	28.52	1658.63
8	0	0	0	93.66	31.66	1753.15
9	1	0	−1	128.20	63.47	1601.37
10	1	0	1	107.00	31.14	1720.50
11	0	1	−1	94.09	53.17	1558.57
12	0	−1	−1	93.61	53.93	1715.86
13	−1	0	1	73.81	34.81	1125.66
14	0	1	1	92.16	26.32	1757.99
15	−1	1	0	54.54	28.46	1002.37

^1^ 2,2′-azino-bis(3-ethylbenzothiazoline-6-sulphonic acid) diammonium salt; ^2^ mg of gallic acid equivalents per g of extract; ^3^ mg of catechin equivalents per g of extract; ^4^ mg Trolox equivalents per mL of extract.

**Table 3:** Corresponding *p*-values for the selected response variable for each obtained coefficient.

Variable	Coefficients	Standard Error	*F*-Value	*p*-Value
**TPH Content**				
Intercept	93.02	5.85		
[T]	20.22	3.58	31.87	0.0024
[t]	0.12	3.58	1.107^−3^	0.9747
[c (HCl)]	−7.20	3.58	4.05	0.1005
[T]^2^	−1.54	5.27	0.085	0.7820
[t]^2^	−12.50	5.27	5.62	0.0638
[c (HCl)]^2^	11.19	5.27	4.50	0.0873
[T]× [t]	−2.24	5.06	0.19	0.6773
[T]× [c (HCl)]	1.66	5.06	0.11	0.7558
[t]× [c (HCl)]	1.18	5.06	0.054	0.8257
**TFL Content**				
Intercept	33.13	3.20		
[T]	−1.84	1.96	0.88	0.3920
[t]	−0.65	1.96	0.11	0.7551
[c (HCl)]	−16.13	1.96	67.69	0.0040
[T]^2^	3.80	2.89	1.73	0.2453
[t]^2^	−7.88	2.89	7.45	0.0413
[c (HCl)]^2^	15.22	2.89	27.92	0.0033
[T]× [t]	1.15	2.77	1.73	0.2453
[T]× [c (HCl)]	3.01	2.77	1.18	0.3267
[t]× [c (HCl)]	−0.35	2.77	0.16	0.9043
**ABTS**				
Intercept	1724.43	82.68		
[T]	300.57	50.63	35.24	0.0019
[t]	−14.97	50.63	0.087	0.7793
[c (HCl)]	−5.66	50.63	0.012	0.9154
[T]^2^	−287.94	74.53	14.93	0.0118
[t]^2^	−85.12	74.53	1.30	0.3051
[c (HCl)]^2^	28.04	74.53	0.14	0.7222
[T]× [t]	−76.60	71.60	1.14	0.3336
[T]× [c (HCl)]	101.02	71.60	1.99	0.2174
[t]× [c (HCl)]	69.57	71.60	0.94	0.3759

**Table 4:** Analysis of variance (ANOVA) of second-order polynomial models for TPH and TFL content and antioxidant activity in *A. millefolium* extracts.

Response	Sum of Squares	DF ^1^	Mean Square	*F*-Value	*p*-Value
TPH content					
Model	4854.44	9	539.38	5.26	0.0411
Residual	512.94	5	102.59		
Lack of fit	441.96	3	147.32	4.15	0.2002
Pure error	70.97	2	35.49		
Total	5367.37	14			
TFL content					
Model	3352.65	9	372.52	12.12	0.0067
Residual	153.72	5	30.74		
Lack of fit	147.08	3	49.03	14.78	0.0640
Pure error	6.63	2	3.32		
Total	3506.37	14			
ABTS					
Model	1.141 × 10^6^	9	1.267 × 10^5^	6.18	0.0295
Residual	1.026 × 10^6^	5	20,518.99		
Lack of fit	97,378.83	3	32,459.61	12.45	0.0753
Pure error	5216.13	2	2608.07		
Total	1.243 ×10^6^	14			

^1^ Degrees of freedom.

**Table 5:** Concentration of hydroxymethylfurfural (HMF) and chlorogenic acid in obtained extracts of *A. millefolium* determined by HPLC.

Run	Temperature	Time	c (HCl)	HMF	Chlorogenic Acid
	[°C]	[min]	[%]	(µg/mL)	(µg/mL)
1	160 (0)	10 (−1)	1.5 (1)	33.4	
2	120 (−1)	20 (0)	0 (−1)	5.8	30.4
3	120 (−1)	10 (−1)	0.75 (0)	19.1	28.4
4	160 (0)	20 (0)	0.75 (0)	39.3	
5	160 (0)	20 (0)	0.75 (0)	33.6	
6	200 (1)	10 (−1)	0.75 (0)	0.0	
7	200 (1)	30 (1)	0.75 (0)	0.0	
8	160 (0)	20 (0)	0.75 (0)	37.0	
9	200 (1)	20 (0)	0 (−1)	20.7	
10	200 (1)	20 (0)	1.5 (1)	0.0	
11	160 (0)	30 (1)	0 (−1)	11.3	
12	160 (0)	10 (−1)	0 (−1)	12.5	16.8
13	120 (−1)	20 (0)	1.5 (1)	28.8	
14	160 (0)	30 (1)	1.5 (1)	33.2	
15	120 (−1)	30 (1)	0.75 (0)	23.5	21.2
